# Cough Recognition Based on Mel-Spectrogram and Convolutional Neural Network

**DOI:** 10.3389/frobt.2021.580080

**Published:** 2021-05-07

**Authors:** Quan Zhou, Jianhua Shan, Wenlong Ding, Chengyin Wang, Shi Yuan, Fuchun Sun, Haiyuan Li, Bin Fang

**Affiliations:** ^1^ Anhui Province Key Laboratory of Special Heavy Load Robot, Anhui University of Technology, Ma’anshan, China; ^2^ Beijing National Research Center for Information Science and Technology, Department of Computer Science and Technology, Tsinghua University, Beijing, China; ^3^ Robotics Institute, School of Automation, Beijing University of Posts and Telecommunications, Beijing, China

**Keywords:** cough recognition, mel-spectrogram, CNN, deep learning, audio, COVID-19

## Abstract

In daily life, there are a variety of complex sound sources. It is important to effectively detect certain sounds in some situations. With the outbreak of COVID-19, it is necessary to distinguish the sound of coughing, to estimate suspected patients in the population. In this paper, we propose a method for cough recognition based on a Mel-spectrogram and a Convolutional Neural Network called the Cough Recognition Network (CRN), which can effectively distinguish cough sounds.

## Introduction

As a disease with a long incubation period and high infection rate, COVID-19 has caused millions of people to be infected and hundreds of thousands of people to died. How to avoid the rapid spread of the epidemic and effectively control the number of infected people has become an urgent issue. Asif et al. found that data from 10,172 COVID-19 laboratory-confirmed cases have shown a correlation with coughing in 54.08% (Sattar Hashmi and Asif, 2020). Therefore, coughing, as a typical symptom of pneumonia, is of great significance in controlling the potential infectious source if it can be quickly and accurately monitored in the population.

Many scholars have studied how to extract features of sound and recognize the sound. Mel Frequency Cepstrum Coefficient (MFCC), as a method of extracting audio features (Shintri and Bhatia, 2015), is widely used in various audio recognition tasks. Xie et al. used MFCC to recognize abnormal voice (Xie et al., 2012). Wang et al. proposed to recognize speech emotion based on improved MFCC (Wang and Hu, 2018). Suksri described a method that used MFCC extracted from the speech signals of spoken words for speech recognition (Ittichaichareon et al., 2012). The Fourier transform (FT) is also widely used in audio processing. Jozef et al. presented a new procedure for the frequency analysis of audio signals (Pucik et al., 2014).

Although these traditional methods are very effective for the extraction of audio features, considering the complexity of the real scene, the method of deep learning may achieve better results. With the development of deep learning, the neural network has played an important role in audio recognition. Oren et al. proposed spectral representations for convolutional neural networks (Rippel et al., 2015). Some LSTM-based networks for speech recognition are also presented (Pundak and Sainath, 2017; Trianto et al., 2018). Compared with traditional methods, deep learning can extract more complex and robust features.

For cough recognition, various methods are proposed. Cough signals are usually obtained by audio or inertial sensors, which can detect the vibration caused by coughing. These sensors include a microphone that can be worn or placed near the user, or a piezoelectric transducer and a high-sensitivity accelerator that can be placed in the throat or chest area (Drugman et al., 2013; Amoh and Odame, 2016; Elfaramawy et al., 2018).

Infante et al. used a machine learning method to recognize dry/wet cough (Infante et al., 2017). Semi-supervized Tree Support Vector Machine is proposed for cough recognition and detection (Hoa et al., 2011). K-NN is also an efficient tool that is often used for cough recognition (Hoyos Barcelo et al., 2017; Vhaduri et al., 2019).

In addition, the Artificial Neural Network (ANN), Gaussian Mixture Model (GMM), Support Vector Machine (SVM), and other methods are also used for cough recognition (Drugman et al., 2011).

The difficulty of cough recognition mainly lies in the distinction of background noise. There are many kinds of sound mixed together in daily scenes. How to effectively distinguish between coughing and other sounds has become a difficult problem to be solved.

In this paper, we propose a cough recognition method based on a Mel-spectrogram and a Convolutional Neural Network (CNN). First, we enhance the audio data and mix the voice in various complex scenes. Then, we preprocess the data to ensure the consistency of data length and convert it into a Mel-spectrogram. At last, we build a CNN-based model to classify the cough using the Mel-spectrogram. At the same time, we make comparisons with some other common methods. After the experiment result comparison, it can be seen that this method can effectively identify and detect coughing in complex scenes. It can be seen that the cough recognition model based on a Mel-spectrogram and a CNN can achieve good results.

## Materials and Methodology

**FIGURE 1:**
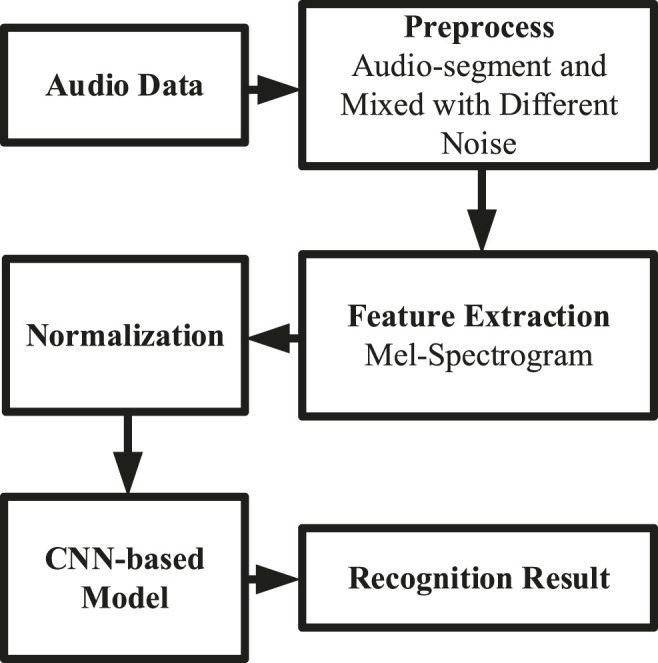
The work-flow diagram.

As shown in Figure 1, the work-flow of our cough classification model is presented.

### Data Augmentation

Considering the natural environment, sound is not produced by a single sound source and the received sound is often the mix of multiple sounds. In order to improve the recognition effect and robustness, we enhance the data, using noise and human voice to mix the cough data.

We selected several audio datasets to make data augmentation, such as the ESC-50 dataset (Piczak, 2015) and the Speech Commands Data Set (Warden, 2018). All cough data comes from the ESC-50 dataset.

Positive Samples 1: Cough. After audio segmentation, we select all cough audio samples as positive samples. We also obtain more cough audio samples by increasing and decreasing the volume.

Positive Samples 2 and 3: Cough + Human Sound and Cough + Natural Sound. In order to enhance the robustness of the model, we also mix cough audio with natural sound (wind, rain, door-clock, footsteps, and other common noises) and human sound (mainly including commonly spoken words such as “go,” “up,” “right,” and so on) respectively as positive samples 2 and 3.

In all the mixed audio, the volume of the coughing sound is adjusted to produce more mixed outcomes of different cough sounds and other sounds.

All of the original and processed cough audio data are labeled as “cough.”

Negative Samples 1: Human Sound. We choose human sounds (mainly include “go,” “up” and, some other common human noises, and all sounds come from different samples which are unused for cough augmentation) from the datasets above as one of the negative samples. So our model can distinguish between cough sounds and human sounds. And all human sounds were mixed with white noise, pink noise, and so on.

Negative Samples 2: Natural Sound: We choose natural noise (wind, rain, pouring-water, footsteps, and other common sounds. All sounds come from different samples which are unused for cough augmentation) from the datasets above as other negative samples.

All human sound and natural sound data are labeled as “others.”

In the end, we have cough sounds, mixed cough audio with natural noise, and mixed cough audio with human sounds as positive samples. At the same time, human sounds and natural sounds are taken as negative samples.

### Data Preprocess

Considering that audio with a too short length of time may make it difficult to recognize the sound, and that audio with a too long length of time may cause the superposition of a variety of uncorrelated sounds, we choose the length of 1 s as the input. And the duration of cough samples in the original dataset is different, so we select the audio containing coughing and divide it into seconds.

#### Mel-Spectrogram

The Mel spectrum contains a short-time Fourier transform (STFT) for each frame of the spectrum (energy/amplitude spectrum), from the linear frequency scale to the logarithmic Mel-scale, and then goes through the filter bank to get the eigenvector, these eigenvalues can be roughly expressed as the distribution of signal energy on the Mel-scale frequency.

After the audio data are processed into 1 s-long data, we transform all the data into Mel-spectrograms so that we can train the convolutional neural networks for recognition.

Audio data usually have complex features, so it is necessary to extract useful features to recognize the audio. The Mel-spectrogram is one of the efficient methods for audio processing and 8 kHz sampling is used for each audio sample.

In the experiment, we employ the Python package called librosa for data processing and all parameters are as follows: (n_fft=1024, hop_length=512, n_mels=128). Then we call the power_to_db function to convert the power spectrum (amplitude square) to decibel (DB) units.

**FIGURE 2:**
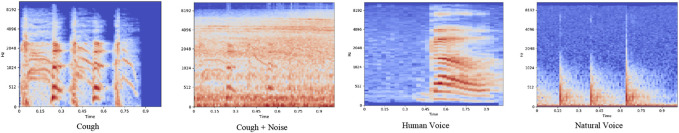
Mel-spectrograms of different voices.

In Figure 2, we show some examples of Mel-spectrograms. As can be seen from the figure, there are some differences in different types of voices. But after mixing noise, some details will be covered, which is helpful for us to test the cough recognition effect of the model for the real scene. And we extract the features of the audio and transform them into feature images, so there are three channels like traditional color images.

#### Normalization

For image input, we normalize them to make the model converge faster. For the Mel-spectrogram, we calculate the mean and standard deviation of the three channels respectively and then normalize them. The normalization formula is as follows:

$$x_{\text{norm}}=\frac{x-\text{mean}\left(x\right)}{\text{std}\left(x\right)},$$ (1)

where *x* denotes the values in different channels and $x_{\text{norm}}$ denotes normalized values.

### Loss Function

The recognition loss function of the model $L_{\text{rec}}$ represents the cross-entropy loss:

$$L_{\text{rec}}=-\frac{1}{n}\sum \left[yln\hat{y}+\left(1-y\right)ln\left(1-\hat{y}\right)\right],$$ (2)

where $\hat{y}$ is the model output, *y* is the true label, and *n* is the number of samples.

### Convolutional Neural Network

With the development of deep learning, more and more deep learning methods are applied to various scenarios, such as image recognition, image classification, speech recognition, machine translation, etc. As a kind of deep learning method, Convolutional Neural Networks (CNN) are widely used in the field of computer vision. In this section, we introduce the components of the proposed CNN-based network.

The convolutional layer is the key of a CNN model, it can effectively reduce the parameters of the model and make it possible for the model to optimize. The calculation formula for the convolutional layer is as follows:

$$x_{j}^{n}=f\left(\sum_{i\in M_{j}}x_{i}^{n-1}\text{*}k_{ij}^{n}+b_{j}^{n}\right),$$ (3)

where $x_{j}^{n}$ is the output feature map, $x_{i}^{n-1}$ is the input feature map, $M_{j}$ is the selected area in the n−1 layer, $k_{ij}^{n}$ is weight parameter, $b_{j}^{n}$ is bias, and *f* is the activation function.

After each convolutional layer, we conduct batch normalization to make the outputs of the convolutional layer stay identically distributed, which can improve the performance of the model. The batch normalization formula is as follows:

$$y_{i}=\gamma \frac{x_{i}-u}{\sqrt{\sigma^{2}+\varepsilon }}+\beta ,$$ (4)

where $x_{i}$ is the output of convolutional layer without activation, *u* is the mean of *x*, $\sigma^{2}$ is the variance of *x*, and γ and β are parameters to learn.

After feature extraction of the convolution layer, although the number of connections between layers has been significantly reduced, the number of neurons in the feature map group has not been significantly reduced. Therefore, like other common models, we add maximum pooling layers to solve this problem.

In the end, we use the fully connected layer as the output layer of the model. The calculation for the fully connected layer is:

$$y_{j}=f\left(\sum_{i=1}^{N}x_{i}\text{*}w_{ij}+b_{j}\right),$$ (5)

where *x* is the input layer, *N* is the number of input layer nodes, $w_{ij}$ is the weight between the links $x_{i}$ and $y_{j}$, $b_{j}$ is the bias, and *f* is the activation function.

### Experiment Approach

The CRN was trained by an Adam optimizer, whose learning rate is 0.0001. The max epoch and batch size were 20 and 64, respectively. The CRN was implemented by Pytorch and trained and tested on a computer with an Intel Core i7-8750H, two 8 GB memory chips (DDR4), and a GPU (Nvidia Geforce GTX 1060 6G).

### Dataset Description

**FIGURE 3:**
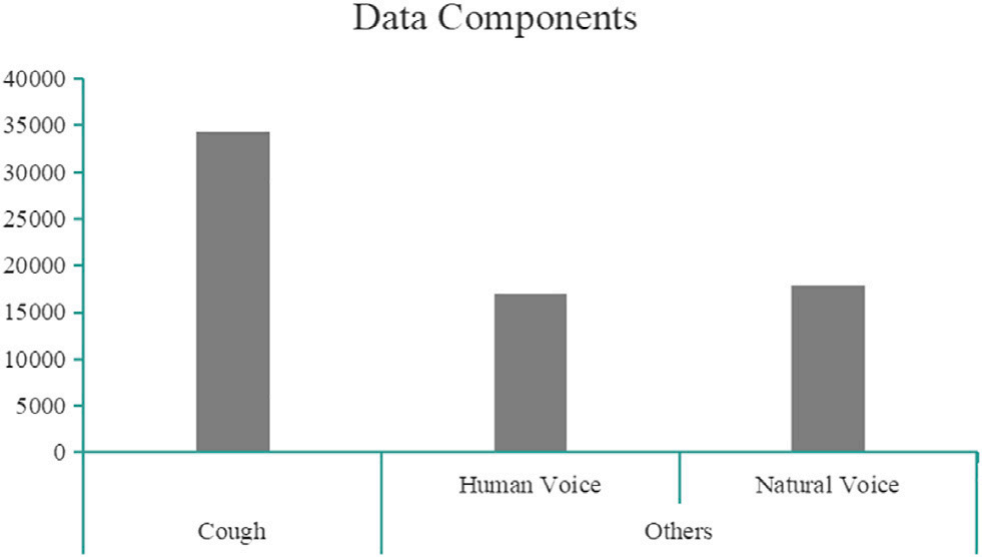
Data components.

Before training, we need to preprocess the audio data. As mentioned in the second part, we obtained 34,320 cough samples augmented by different audio data, including 17,160 cough + human sound samples, 17,160 cough + natural sound samples, 17,050 human sounds, and 17,919 different noises. As shown in Figure 3, data components have been provided. In order to evaluate the model better, we use two ways to divide the processed dataset.

#### Random Division Dataset

After all data are processed, 80% are randomly selected as the training set, 10% as the verification set, and 10% as the test set. Considering that due to data augmentation, some data may leak the features of coughing.

#### No-Leakage Division Dataset

After all data are processed, we select almost 80% which we augment as the training set and 10% is augmented from completely different cough audio as the test set. In this way, the cough sounds of the training and test sets come from different original data, so that we can evaluate the generalization ability of the model.

After all data are split, the mean and variance of each channel are calculated. They are normalized to make the model converge better.

### Performance Measurements

In order to better evaluate the performance of the model, we list several indicators used to evaluate the model.

#### Accuracy

The indicator that the samples with a correct reaction classification account for the total samples.

#### Recall

The ratio of the number of samples recognized correctly to the total number of samples recognized.

#### Precision

The ratio of the number of samples recognized correctly to the number of samples that should be recognized.

#### F1 Score

It is an index used to measure the accuracy of the binary classification model.

$$\text{Accuracy}=\frac{\text{TP}+\text{TN}}{\text{TP}+\text{TN}+\text{FP}+\text{FN}},$$ (6)

$$\text{Recall}=\frac{\text{TP}}{\text{TP}+\text{FN}},$$ (7)

$$\text{Precision}=\frac{\text{TP}}{\text{TP}+\text{FP}},$$ (8)

$$\text{F}1\text{ Score}=\frac{2*\text{Precision}*\text{Recall}}{\text{Recall}+\text{Precion}},$$ (9)

where TP (True Positive) denotes samples of coughing that are correctly recognized by the model. FP (False Positive) which denotes samples of coughing that are recognized as others by the model. TN (True Negative) which denotes samples of others that are correctly recognized by the model. FN (False Negative) which denotes samples of others that are incorrectly recognized as coughing by the model.

### Experiment Based on Mel-Spectrogram + CNN

**FIGURE 4:**
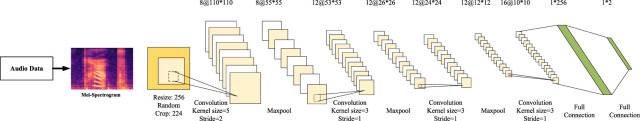
The Architecture of the Mel-spectrogram and CNN model.

The Mel-spectrogram is an effective tool to extract hidden features from audio and visualize them as an image. A CNN model can effectively extract features from images, and then complete tasks such as classification and recognition. Therefore, we use the CNN model to effectively classify the audio and to realize the accurate recognition and detection of coughing. In Figure 4, the architecture of this model has been illustrated.

Considering the different positions of coughing in audio, the relative positions of coughing are also different. Before we feed the image into the network, we first unify the image size into 256 × 256, and then randomly select 224 × 224 size parts for the recognition of different cough positions.

## Results

After two methods of dataset division and training, we get the performance of the cough recognition task.

### Experiment on Random Division Dataset

**FIGURE 5:**
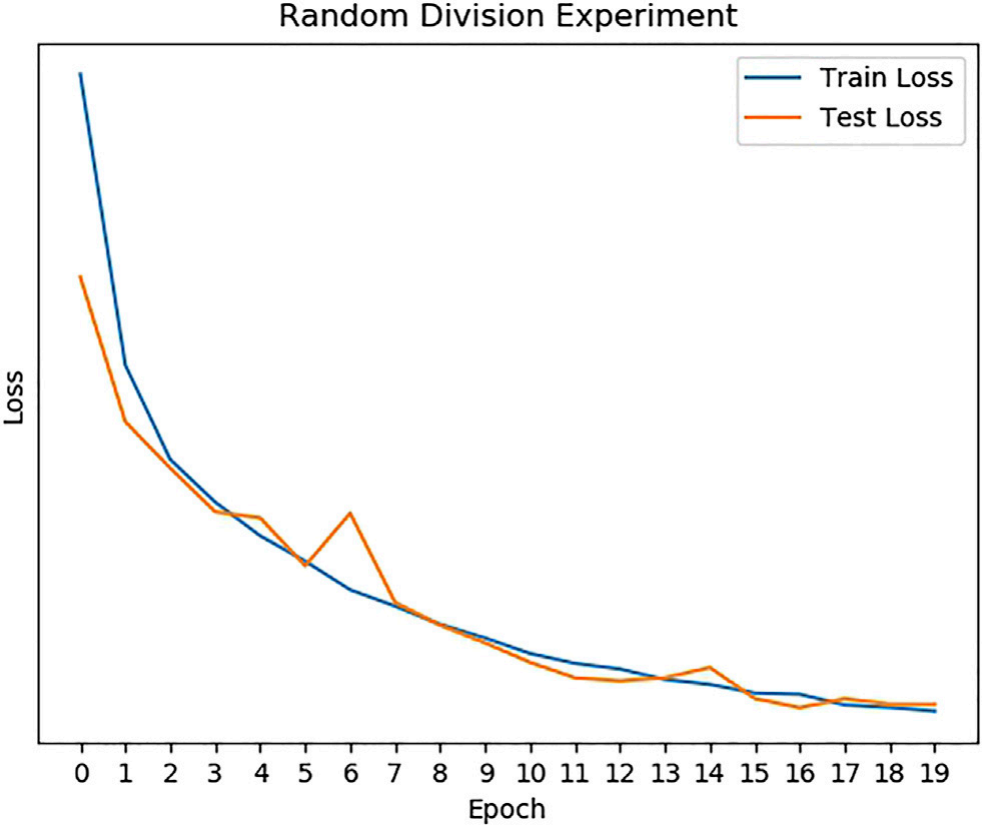
The loss of the random division experiment.

As shown in Table 1, we can find that Mel-Spectrogram + CNN can achieve the best performance in cough recognition than other methods. For randomly divided datasets, the correct recognition rate is 98%. It can be seen that the model can still achieve good recognition performance even if a variety of different sounds are mixed. The train/test loss curves are presented in Figure 5.

**TABLE 1:** The comparison results of different methods.

Methods	Random division recognition task				No-leakage division recognition task			
	Accuracy (%)	Recall (%)	Precision (%)	F1 Score (%)	Accuracy (%)	Recall (%)	Precision (%)	F1 Score (%)
Mel-spectrogram + CNN	98.18	99.18	99.28	99.23	95.18	93.33	100	96.55
Mel-spectrogram + BP	94.34	87.50	100	93.33	91.44	93.75	93.75	93.75
MFCC + CNN	97.43	88.88	100	94.12	94.04	100	88.88	94.11
MFCC + BP	96.12	97.19	93.87	97.19	93.45	90.91	100	95.23
MFCC + SVM	95.76	96.99	94.57	95.77	93.29	93.56	91.79	92.67
MFCC + K-means	52.93	42.86	53.09	47.43	50.34	42.44	44.96	43.66
MFCC + Naive-bayes	88.57	95.31	83.83	89.20	78.81	82.43	73.87	77.92
MFCC + LightGBM	95.73	98.46	93.29	95.80	89.89	88.17	89.38	88.77

### Experiment on No-Leakage Division Dataset

Considering that the model needs to cope with the cough sounds of different people, we add an experiment to estimate the generalization ability of the model. In this experiment, all the cough data are augmented, but the cough sound in the training set and the test set come from totally different collection objects. In this way, it can detect whether the model has the ability to recognize the cough sound produced by strange sound sources effectively.

**FIGURE 6:**
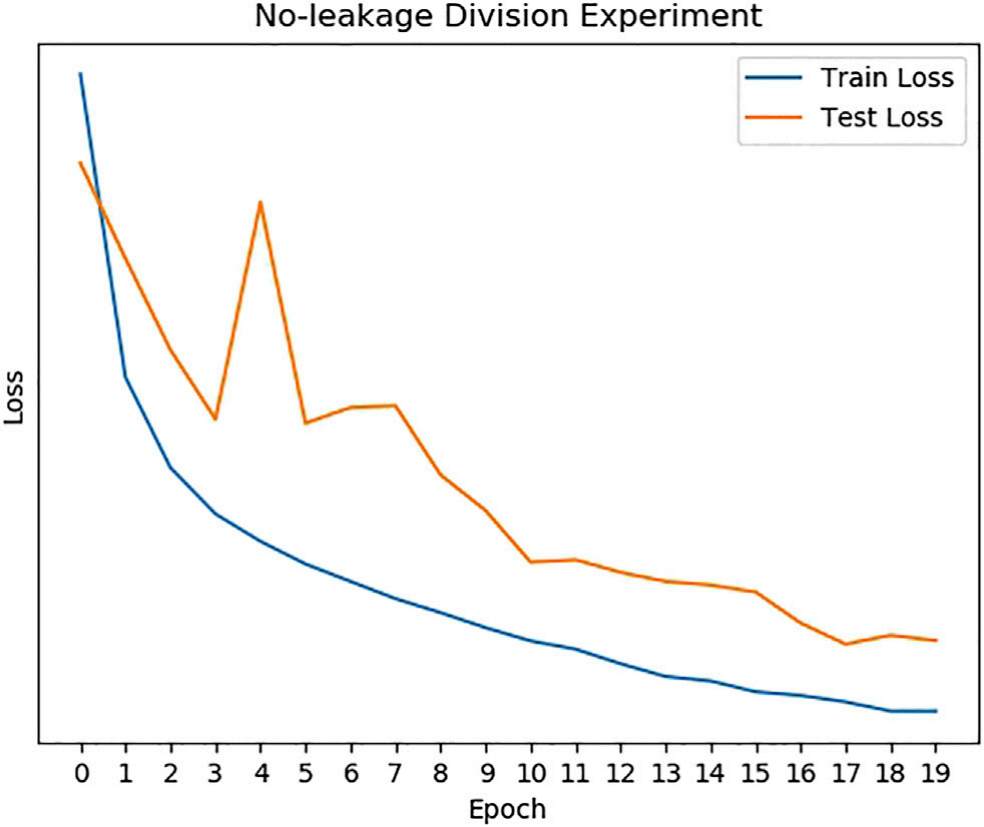
The loss of the no-leakage division experiment.

The train/ test loss curves of no‐ leakage experiment are presented in Figure 6 and the experiment result is shown in Table 1. The no-leakage recognition accuracy is 95.18% and the F1 score is the highest of all methods. It can be seen that the model performs well during generalization cough recognition tasks.

### Experiment Based On Other Traditional Methods

In order to prove the effectiveness of this method, we use several other methods for comparison.

#### MFCC

MFCC is an effective method to extract audio features. We use this method to preprocess the original audio data and then pass it to the different model. In order to make it suitable for the linear model, in the experiment, we take the average value on each dimension.

#### Back Propagation Network

BP is a multilayer feedforward network which has a strong nonlinear mapping ability. In our experiment, we build a four-layer BP neural network and the activation is ReLU.

#### Support Vector Machine

A Support Vector Machine (SVM) is a kind of generalized linear classifier that classifies data according to supervised learning.

#### K-Means

The K-means algorithm is an iterative clustering algorithm. Firstly, it randomly selects *K* objects as the initial clustering center. Then it calculates the distance between each object and each seed cluster center and assigns each object to the nearest cluster center.

#### Naive-Bayes

Naive Bayes is a classification method based on Bayes theorem and the independent hypothesis of characteristic conditions.

#### LightGBM

LighGBM is one of the boosting set models. It is an efficient implementation of the Gradient Boosting Decision Tree (GBDT) as XGBoost. In principle, it is similar to GBDT and XGBoost. It uses the negative gradient of loss function as the residual approximation of the current decision tree to fit the new decision tree.

All results based on these methods are shown in Table 1, and we can find that the CNN model is better than these methods in recognition accuracy and other indicators.

## Conclusion

In this work, we proposed a cough recognition network (CRN) based on the CNN model and a Mel-spectrogram. From the experiments result based on random division and no-leakage division datasets, we can find that the proposed CRN can achieve excellent performance in cough recognition. Compared to other methods, the accuracy of CRN is highest and most of the indexes are the best. In order to estimate the generalization ability of the model, we have collected some cough sounds that were not included in training. We find that the CRN can also recognize them efficiency. Experiments show that the model can recognize coughing in complex scenes effectively, and can recognize coughing with various other sounds correctly, which is good for cough monitoring in daily life. Cough recognition is a potential solution for disease management during the COVID-19 pandemic and reduces epidemic prevention workers’ exposure possibility.

Although the model has achieved good recognition results, there are still some problems that need to be further solved. For example, the audio length is now limited to 1 s. When the intercept position is not right, it may be misjudged.

## Data Availability

Publicly available datasets were analyzed in this study. This data can be found here: https://github.com/karolpiczak/ESC-50 ESC-50 Dataset http://download.tensorflow.org/data/speech_commands_v0.02.tar.gz Speech Commands Dataset.
